# Depressive Symptoms and Their Associated Factors in Nursery School Teachers: A Multicenter Cross-Sectional Study

**DOI:** 10.7759/cureus.16545

**Published:** 2021-07-21

**Authors:** Satiko Kataoka, Kana Kitamura, Yuki Kataoka, Yosuke Yamamoto

**Affiliations:** 1 Diabetes and Endocrinology, Kyoto University, Kyoto, JPN; 2 Psychology, Ayaha Corporation, Kusatsu, JPN; 3 Internal Medicine, Kyoto Min-iren Asukai Hospital, Kyoto, JPN; 4 Epidemiology and Public Health, Kyoto University, Kyoto, JPN

**Keywords:** depressive symptoms, nursery school teacher, mental health inventory-5, job content questionnaire, work limitation questionnaire

## Abstract

Background

Although nursery school teachers may experience depressive symptoms, there have been few studies exploring the associated factors. The objective of this study was to determine the prevalence of depression and explore its associated factors in nursery school teachers.

Methods

This cross-sectional study surveyed nursery school teachers in Sakyo-ku, Kyoto City to determine the prevalence of depressive symptoms as measured by the five-item Mental Health Inventory. We used a logistic regression model to assess the factors.

Results

Respondents were 148 teachers (36%) out of 410 nursery school teachers in 21 nursery schools, and 65 (44%) indicated that they had depressive symptoms. Using the Work Limitation Questionnaire (WLQ), productivity loss score (adjusted risk ratio [ARR], 1.17; 95% confidence interval [95% CI], 1.02 to 1.34) and psychological demands (ARR, 1.25; 95% CI, 1.02 to 1.53) were found to be associated with depressive symptoms.

Conclusions

The associated factors with depressive symptoms were high psychological demands and a high degree of presenteeism. Further prospective cohort studies with larger sample sizes should be conducted to confirm these relationships.

## Introduction

Mental health is an important occupational health issue that can lead to significant expenses for employers, and mental health issues can lead to absences from work [1]. In a previous report, approximately 40% of employees showed depressive symptoms [2,3]. Mental health issues are prevalent among nursery school teachers in particular [4], as it has been reported that approximately 45% of them have depressive symptoms [5]. 

The associated factors for depressive symptoms arising from work are high psychological demands, low social support from supervisors and coworkers, low decision latitude, and high degrees of presenteeism [6,7]. However, there have been few studies on depressive symptoms among nursery schoolteachers, and there are very few reports on the associated factors for depressive symptoms in nursery school teachers [5]. This study aimed to determine the prevalence of depressive symptoms and explore its associated factors among nursery school teachers. 

## Materials and methods

This cross-sectional study surveyed nursery school teachers from 21 nursery schools in Sakyo-ku Hoikushikai, a branch of Zenkoku Hoikushikai, which is the largest association of nursery school teachers in Japan to promote the welfare of children and their parents [8]. The recruiting process was described in the previous report [8]. We explained our survey in Sakyo-ku Hoikushikai, and 21 nursery schools out of 27 agreed to participate in the survey.

The nursery school teachers voluntarily participated. Each nursery school teacher answered an anonymous questionnaire. Individual informed consent was obtained from all the participants. The data collection period ranged from October 2017 to March 2018.

Outcomes

The main results of these surveys were the presence of depressive symptoms. Mental health was measured using the five-item Mental Health Inventory (MHI-5), whose cut-off of severe depressive symptoms was 52 [9]. The MHI-5 is derived from the mental health domain of the Short Form (SF) 36 Health Survey [10]. Teachers with scores of ≤ 52 were considered to have depressive symptoms.

Exposures

We chose five factors as the possible causes or leading factors for depressive symptoms, as these were identified in previous reports and panel discussions involving epidemiologists, psychologists, industrial physicians, and nursery school teachers. The factors all described the degree of presenteeism, psychological demands, social support (supervisors and coworkers), decision latitude, and age [6,7]. We measured the degree of presenteeism using the productivity loss score of the Work Limitation Questionnaire (WLQ) [11,12]. WLQ is a 25-item self-administered questionnaire that measures the degree to which health problems interfere with job performance in the prior two weeks. The WLQ consists of four domains: time management, physical demands, mental-interpersonal demands, and output demands. The scale score for these four domains ranges from 0 to 100 and indicates the percentage of time that nursery school teachers were limited in each domain. The time management scale reflects difficulties in performing work on time and scheduling tasks. The physical scale reflects personal disabilities into performing job tasks that require bodily strength, movement, endurance, coordination, and flexibility. The mental-interpersonal scale reflects the difficulties in performing cognitive job tasks and tasks using sensory information and interacting with people on the job. The output scales measure decrements in personal disability to complete work on time and meet demands for quantity, quality, and timeliness of completed work. Using these four domains, the WLQ productivity loss score was calculated. The WLQ productivity loss score indicates the percentage of impairment in work output due to health problems ranging from 0 to 24.9%, which was calculated using the original formula [13]. Psychological demand, social support, and decision latitude were measured using 22 items selected from the Job Content Questionnaire (JCQ) [14,15]. The JCQ scale is frequently used to predict job-related stress regarding psychological demand, social support, decision latitude, and others. The range for psychological demand, social support, and decision latitude were 12-48, 8-32, and 24-96, respectively in this study. A higher psychological demand score indicates greater psychological demands, a higher decision latitude score indicates that teachers have higher skill discretion and decision authority, and a higher score on social support indicates greater support from supervisors and coworkers.

Characteristics of participants

Data collected included sex, relationship status, years of service, work status (full-time employee/part-time employee), duty hours, break times, smoking status (smoker/non-smoker), comorbidities, history of depression, and the four WLQ domains. Some of the participants had worked in their position for less than one year, so service is expressed in years and months.

The Japanese Labor Standard Act states that rest times must be at least 45 minutes during duty hours when more than six hours are worked per day, and at least one hour when more than eight hours are worked per day [16]. Break times are therefore expressed in hours and minutes per day. We collected the data of total number of all the teachers in 21 nursery schools, from Kyoto City’s open data available on the web [17].

Statistical analysis

The median and interquartile range (IQR) were used for continuous variables and number and percentage for categorical variables for the descriptive analysis. For the univariate analysis, the Wilcoxon rank-sum test was used for continuous variables, and Fisher’s exact test was used for categorical variables. In the multivariable analysis, we used a logistic regression model to examine five factors associated with depressive symptoms. The dependent variables were age (years), WLQ productivity loss score (percentage), psychological demands, social support, and decision latitude. We converted the three scores of the JCQ into z-scores and the odds ratio into a risk ratio.

Complete case analysis was used, except for MHI-5, for the domains of WLQ, WLQ productivity loss score, service years, and break time. For MHI-5 and WLQ, we imputed scores for missing values, as indicated by the manuals for each questionnaire [13,18]. Regarding service years and break time, we handled the missing data in two ways. First, with regard to service years, if participants responded only in years or months, we regarded no response as 0, and the same was done for break time (hours/minutes). If respondents did not supply answers for both years or months regarding years of service or hours or minutes regarding break time, this was considered as missing data. Second, we also described the data without imputation for service years and break times. If respondents answered only with years or months for years of service, or only hours or minutes for break times, we regarded the variables as missing. We used Stata® 14.2 (StataCorp, College Station, TX, USA) for all analyses.

## Results

Out of the 410 nursery school teachers from 21 nursery schools, 154 (38%) answered the questionnaire, and 148 (96%) out of 154 also answered the MHI-5. Therefore, the total response rate was 36%. We excluded six responses that did not answer the MHI-5. The participants’ characteristics are shown in Table 1. The median age was 40.5, and only 10 (7%) were male. In total, 44% (65) of teachers showed evidence of depressive symptoms. These teachers were on average younger than the other 56% (median [IQR], 36 [28 to 46] vs. 44 [33 to 53]). Compared to nursery school teachers without depressive symptoms, the scores of those with depressive symptoms were higher in regard to time management (median [IQR], 16.7 [5.0 to 25.0] vs. 10.0 [0.0 to 20.0]), mental-interpersonal demands (median [IQR], 25.0 [16.7 to 33.3] vs. 16.7 [8.3 to 25.0]), output demands (median [IQR], 22.5 [10.0 to 35.0] vs. 15.0 [2.5 to 25.0]), WLQ productivity loss (median [IQR], 6.4 [4.3 to 8.4] vs. 4.6 [2.8 to 6.3]), psychological demands (median [IQR], 38 [35 to 41] vs. 33 [31 to 37]), and decision latitude (median [IQR], 72 [66 to 78] vs. 68 [62 to 76]).

**Table 1:** Participant’s characteristics IQR, interquartile range. MHI-5, the five-question Mental Health Inventory. WLQ, the Work Limitation s Questionnaire. JCQ, the Job Content Questionnaire. †Wilcoxon rank-sum test, ‡Fisher’s exact test

	Total (n=148)	MHI-5 > 52 (n=83)	MHI-5 ≤ 52 (n=65)	Missing	P-value
Age (years), median, [IQR]	40.5 [31 to 51.5]	44 [33 to 53]	36 [28 to 46]	0	0.008†
Male, n (%)	10 (7%)	4 (5%)	6 (9%)	1 (1%)	0.330‡
Teacher living with his or her partner n (%)	75 (51%)	45 (54%)	30 (46%)	0	0.410‡
Years of service (years), median, [IQR]	13.4 [5.8 to 19.8]	15.0 [6.8 to 24.0]	12.0 [3.9 to 18.0]	0	0.100†
Full-time employee, n (%)	112 (76%)	58 (70%)	54 (83%)	0	0.082‡
Duty hours (per week) median, [IQR]	45.0 [38.0 to 53.0]	44.0 [38.0 to 50.0]	45.0 [40.0 to 55.0]	4 (3%)	0.330†
Break time (minutes per day) median, [IQR]	45 [30 to 60]	50 [30 to 60]	45 [20 to 60]	4 (3%)	0.100†
Smoking, n (%)	22 (15%)	12 (15%)	10 (15%)	1 (1%)	1.000‡
Comorbidities					
Body pain, n (%)	101 (68%)	51 (61%)	50 (77%)	0	0.052‡
History					
Depression, n (%)	7 (5%)	3 (4%)	4 (6%)	9 (6%)	0.700‡
Malignancy, n (%)	4 (3%)	2 (2%)	2 (3%)	9 (6%)	1.000‡
WLQ					
Time management (1 to 100) median, [IQR]	12.5 [5.0 to 25.0]	10.0 [0.0 to 20.0]	16.7 [5.0 to 25.0]	7 (5%)	0.041†
Physical demands (1 to 100) median, [IQR]	20.4 [0.0 to 37.5]	18.3 [0.0 to 41.7]	22.9 [0.0 to 33.3]	4 (3%)	0.980†
Mental-interpersonal demands (1 to 100) median, [IQR]	21.7 [11.1 to 30.6]	16.7 [8.3 to 25.0]	25.0 [16.7 to 33.3]	4 (3%)	<0.001†
Output demands (1 to 100) median, [IQR]	15.0 [5.0 to 30.0]	15.0 [2.5 to 25.0]	22.5 [10.0 to 35.0]	6 (4%)	0.004†
WLQ productivity loss score (0 to 24.9) (%) median, [IQR]	5.2 [3.3 to 7.3]	4.6 [2.8 to 6.3]	6.4 [4.3 to 8.4]	10 (7%)	<0.001†
JCQ					
Psychological demand (12 to 48) median, [IQR]	36 [32 to 39]	33 [31 to 37]	38 [35 to 41]	3 (2%)	<0.001†
Social support (8 to 32) median, [IQR]	24 [22 to 28]	24 [22 to 27]	24 [22 to 28]	3 (2%)	0.700†
Decision latitude (24 to 96) median, [IQR]	70 [64 to 76]	68 [62 to 76]	72 [66 to 78]	3 (2%)	0.019†

The results of the multivariable logistic regression model are presented in Table 2. The WLQ productivity loss score (adjusted risk ratio [ARR], 1.17; 95% confidence interval [CI], 1.02 to 1.34) and psychological demands (ARR, 1.25; 95% CI 1.02 to 1.53) were associated with depressive symptoms. As for years of service, 44 (30%) did not complete the questions, and for break time, 105 (71%) did not complete the questions (refer to Table 3 in the Appendices).

**Table 2:** Factors associated with depressive symptoms (n=133) CI, confidence interval. WLQ, the Work Limitation s Questionnaire.

Factors associated with depressive symptoms	Crude risk ratio	95%CI	Adjusted risk ratio	95%CI
Age (years)	0.99	0.99 to 0.99	0.99	0.99 to 1.00
WLQ productivity loss score (per percent)	1.20	1.05 to 1.38	1.17	1.02 to 1.34
Psychological demand (per point)	1.42	1.19 to 1.69	1.25	1.02 to 1.53
Social support (per point)	1.03	0.85 to 1.24	0.98	0.79 to 1.21
Decision latitude (per point)	1.20	1.01 to 1.42	1.18	0.98 to 1.41

## Discussion

Of the 148 nursery school teachers, 65 (44%) indicated depressive symptoms. In the multivariable analysis, a high degree of psychological demands and presenteeism were associated with these symptoms.

Regarding psychological demands, our results replicated those of a previous systematic review including cohort studies, which suggested that psychological demands are associated with depressive symptoms in other occupations [6].

The present study was the first to describe and examine the degree of presenteeism using WLQ in nursery school teachers. In the univariate analysis, the percentage of time in the loss of time management, mental-interpersonal demands, and output demands, and the WLQ productivity loss score were higher in nursery school teachers with depressive symptoms than nursery school teachers without symptoms. Nursery school teachers must be talented planners and need to cooperate with their coworkers and students’ parents [19]. Therefore, the qualities included in the three domains are important for them. Calculating the degree of presenteeism in nursery school teachers with or without depressive symptoms is important for management. Regarding The WLQ productivity loss score, nursery school teachers’ scores were higher than those reported for Japanese individuals with chronic symptoms, chosen by stratified random sampling [12]. Another report revealed that nursery school teachers usually retire early, and one of the main reasons is health problems [20]. These findings suggest that some interventions to help with health problems, including mental health, are needed for nursery school teachers.

In the multivariable analysis, the WLQ productivity loss score calculated from the four domains was also higher in nursery school teachers with depressive symptoms than those without symptoms. It was previously reported in other occupations that presenteeism is an associated factor with depressive symptoms [7,21]. As this was a cross-sectional study, a prospective cohort study is required to clarify the causal relationship in nursery school teachers. In addition, when a decrement in work output is detected in nursery school teachers, depressive symptoms should be considered as an important cause.

As for the other three factors, associations with depressive symptoms were not detected in this study. However, this may be due to the small sample size. Therefore, further studies with larger sample sizes are required. We conducted this study in Kyoto City, and our results may not be applicable to other regions. Therefore, studies in other regions are also required.

The strength of the present study is that it was a multicenter study using the validated MHI-5, JCQ, and WLQ scales. However, this study has several limitations. First, the percentage of responses was only 38%, and selection bias may have taken place. This may be because teachers were not interested in completing the survey, and the overall prevalence of depressive symptoms in nursery school teachers may be lower. Additionally, missing data in some variables, such as break time and years of service, were prevalent. Most of the teachers with missing data had partly answered the questions regarding break time and years of service. We did not use these variables (break time and years of service) in the multivariable analysis, and described both imputed and raw data for such variables. Therefore, the bias from missing data did not largely affect our multivariable analysis or interpretation. This bias could have increased for break times, considering most nursery school teachers used minutes, and not hours, to answer this question. In years of service, the proportion of missing data may have resulted in lower years of service overall. Most of the missing data were months component, and nursery school teachers with over one year of experience tended to answer in years. Third, this was a cross-sectional study, and the reverse causal relationship between depressive symptoms and their associated factors could not be examined. Finally, the sample size of this study was small, and an analysis using a multilevel multivariable logistic regression model could not be performed.

## Conclusions

In this study, we revealed the prevalence of depressive symptoms among nursery school teachers. In the multivariable analysis, depressive symptoms were associated with high psychological demands and a high degree of presenteeism. However, further prospective cohort studies with larger sample sizes are required to provide more evidence.

## Appendices

**Table 3:** Data of years of service (years) and break time without complement IQR, interquartile range. MHI-5, the five-question Mental Health Inventory. †Wilcoxon rank-sum test

	Total (n=148)	MHI-5 > 52 (n=83)	MHI-5 ≤ 52 (n=65)	Missing	P-value
Years of service (years), median, [IQR]	12.8 [4.8 to 19.8]	15.0 [6.8 to 24.8]	10.3 [3.8 to 17.1]	44 (30%)	0.039†
Break time, median, [IQR]	60 [60 to 60]	60 [60 to 60]	60 [45 to 60]	105 (71%)	0.790†
